# Oil palm vegetation liquor: a new source of phenolic bioactives

**DOI:** 10.1017/S0007114511002121

**Published:** 2011-06-06

**Authors:** Ravigadevi Sambanthamurthi, YewAi Tan, Kalyana Sundram, Mahinda Abeywardena, T. G. Sambandan, ChoKyun Rha, Anthony J. Sinskey, Krishnan Subramaniam, Soon-Sen Leow, Kenneth C. Hayes, Mohd Basri Wahid

**Affiliations:** 1 Malaysian Palm Oil Board, 6, Persiaran Institusi, Bandar Baru Bangi, 43000 KajangSelangor, Malaysia; 2 Malaysian Palm Oil Council, 2nd Floor, Wisma Sawit, Lot 6, SS6, Jalan Perbandaran, 47301 Kelana Jaya, Selangor, Malaysia; 3 Commonwealth Scientific and Industrial Research Organisation, Gate 13, Kintore Avenue, Adelaide, SA5000, Australia; 4 Massachusetts Institute of Technology, 77 Massachusetts Avenue, Cambridge, MA02139, USA; 5 MAHSA University College, Jalan University Campus, 59100Kuala Lumpur, Malaysia; 6 Brandeis University, 415 South Street, Waltham, MA02454, USA

**Keywords:** Oil palm phenolics, Caffeoylshikimic acid, Antioxidant activity, Teratology

## Abstract

Waste from agricultural products represents a disposal liability, which needs to be addressed. Palm oil is the most widely traded edible oil globally, and its production generates 85 million tons of aqueous by-products annually. This aqueous stream is rich in phenolic antioxidants, which were investigated for their composition and potential *in vitro* biological activity. We have identified three isomers of caffeoylshikimic acid as major components of oil palm phenolics (OPP). The 2,2-diphenyl-1-picrylhydrazyl assay confirmed potent free radical scavenging activity. To test for possible cardioprotective effects of OPP, we carried out *in vitro* LDL oxidation studies as well as *ex vivo* aortic ring and mesenteric vascular bed relaxation measurements. We found that OPP inhibited the Cu-mediated oxidation of human LDL. OPP also promoted vascular relaxation in both isolated aortic rings and perfused mesenteric vascular beds pre-contracted with noradrenaline. To rule out developmental toxicity, we performed teratological studies on rats up to the third generation and did not find any congenital anomalies. Thus, these initial studies suggest that OPP is safe and may have a protective role against free radical damage, LDL oxidation and its attendant negative effects, as well as vascular constriction in mitigating atherosclerosis. Oil palm vegetation liquor thus represents a new source of phenolic bioactives.

Reactive oxygen species such as superoxide anions, H_2_O_2_ and hydroxyl radicals may contribute to the genesis of CHD, diabetes, cancer and other degenerative diseases^(^ ^1^ ^–^ ^5^ ^)^. Many epidemiological studies have indicated that consumption of fruit and vegetables decreases the risk of degenerative diseases^(^ ^6^ ^,^ ^7^ ^)^, and that the beneficial effects, in part, can be ascribed to the antioxidant activities of minor phytochemical components, including phenolic compounds^(^ ^6^ ^–^ ^10^ ^)^.

The oil palm (*Elaeis guineensis*) from the family Arecaceae is a high oil-producing tropical plant that appears to have an effective antioxidative component to counter the oxidative stress exerted by high temperature and intense sunlight. Indeed, the oil palm is a rich source of phytochemicals^(^ ^11^ ^–^ ^13^ ^)^. While the technology for recovery of fat-soluble antioxidants such as tocopherols, tocotrienols and carotenoids from palm oil is well established^(^ ^11^ ^)^, it is only recently that the technology for harvesting water-soluble antioxidants from oil palm has become available^(^ ^12^ ^,^ ^14^ ^–^ ^17^ ^)^.

During the palm oil milling process, water-soluble phenolics are discarded in the waste stream, amounting to 85 million tons per year globally. A recovery procedure for oil palm phenolics (OPP) has been developed to isolate concentrations^(^ ^14^ ^)^ suitable for biological applications, providing an opportunity to transform a bioburden into a range of potential applications for health and wellness.

Based on growing evidence that plant phenolics are beneficial to health, OPP was assessed for positive bioactivities. In the present study, the chemical constituents and composition of specific OPP are described. In addition, *in vitro* antioxidant and LDL oxidation experiments, as well as *ex vivo* aortic ring and mesenteric vascular bed experiments, were designed to identify the potential bioactivities of OPP. We also carried out teratological studies to assess whether OPP is safe for consumption.

## Materials and methods

### HPLC, MS and NMR analyses of oil palm phenolics

OPP obtained according to the methods described by Sambanthamurthi *et al.* ^(^ ^14^ ^)^ was subjected to separation by reversed-phase HPLC, and the individual peaks were characterised by MS and NMR spectroscopy. A freeze-dried OPP sample (10 mg) was dissolved in 1 ml of internal standard citric acid solution (α-cyano-hydroxycinnamic acid (1 mg/ml) in 0·2 % (v/v) citric acid). The OPP sample was then extracted by adding 1 ml of ethyl acetate, shaking the solution well and then allowing it to settle. The supernatant (200 μl) was evaporated and reconstituted with 200 μl of 0·2 % (v/v) citric acid. This reconstituted solution was then injected into an analytical HPLC system. Each compound in the sample was determined using the peak ratio of the compound *v.* the internal standard. The calibration curve was used to obtain the concentration of each compound based on their peak ratios.

Samples were analysed on a Hitachi system comprising a low-pressure mixing pump (model L7100; Hitachi, Richmond, CA, USA), an autosampler (model L7200; Hitachi), a photodiode detector (L7450; Hitachi) and D-7000 HPLC system software for integration (Hitachi). Chromatographic separation was achieved using a 250 × 4·0 mm reversed-phase column (GL Exsil ODS 5 μm inner diameter) (SGE Inc., Austin, TX, USA). The mobile phase used was a binary gradient system, with phase A comprising 10 mm-sodium sulphate containing 0·02 % (v/v) phosphoric acid (pH 2·75) and phase B comprising methanol–acetonitrile (70:30, v/v). Sample injection volume was 10 μl and a flow rate of 0·8 ml/min was used. The gradient elution with a total run time of 60 min was as follows: started from 95 % (v/v) solvent A and 5 % (v/v) solvent B, increased to 35 % (v/v) solvent B over 45 min, then increased to 100 % (v/v) solvent B over 3 min, then maintained at 100 % (v/v) solvent B for 2 min and finally decreased to 5 % (v/v) solvent B over 10 min. A MALDI Voyager (Applied Biosystems, Foster City, CA, USA) was used to determine the molecular weights of these compounds. ^1^H NMR and ^13^C NMR were carried out with a Varian Inova600 (600 MHz) equipped with a 5 mm inner diameter probe (Varian, Inc., Walnut Creek, CA, USA).

### Analysis of oil palm phenolics antioxidant activity

The phenolic content of OPP used in animal studies was determined using the Folin–Ciocalteu reagent^(^ ^18^ ^)^. The free radical scavenging activity was assessed using the 2,2-diphenyl-1-picrylhydrazyl (DPPH) reagent^(^ ^9^ ^)^.

### Copper-mediated LDL oxidation

To evaluate the ability of OPP to inhibit Cu-mediated LDL oxidation, conjugated dienes were continually monitored at 5 min intervals at 37°C by UV absorption at 234 nm. LDL was prepared as described by Sundram *et al.* ^(^ ^19^ ^)^. LDL oxidation was initiated by the addition of copper sulphate at a final concentration of 6 μmol–90 μg of LDL-cholesterol in a final volume of 1 ml. Purified catechin was used as a control phenolic compound. The purified test compounds (catechin obtained from Sigma Chemical, St Louis, MO, USA) and OPP extracts were added immediately before the addition of the oxidant. All LDL oxidations were performed in triplicates. The lag time in the presence or absence of the test compounds was determined as the intercept of the slopes for the lag and propagation phases. This was compared with the control oxidised LDL to determine the percentage LDL oxidation inhibition.

### Aortic ring and mesenteric vascular bed preparation for vascular studies

The use of animals in the present study was approved by the Commonwealth Scientific and Industrial Research Organisation-Health Sciences and Nutrition Animal Experimentation Ethics Committee. All experimental procedures including the care, handling and maintenance of the experimental animals were performed according to the National Health and Medical Research Council guidelines for the use and care of animals for experimental purposes.

Isolated segments (3 mm) of the thoracic aorta from male normotensive Wistar Kyoto and spontaneously hypertensive rats (12–14 weeks old) supplied by the Animal Resources Centre (Canning Vale, WA, Australia) were mounted under isometric conditions in 15 ml organ bath chambers containing physiological Krebs–Henseleit solution (113 mm-NaCl, 4·8 mm-potassium chloride, 1·2 mm-KH_2_PO_4_, 1·2 mm-MgSO_4_, 25 mm-NaHCO_3_, 2·5 mm-CaCl_2_, 11·2 mm-glucose and 0·57 mm-ascorbic acid in Milli Q-treated water), bubbled with carbogen and maintained at 37°C as described in detail previously^(^ ^20^ ^,^ ^21^ ^)^. In some rings, the endothelium was removed by careful rubbing of the intima with a moistened cotton swab. The tissues were equilibrated for at least 60 min before contracting with potassium chloride (20 mmol/l) to test tissue viability. The rings were pre-contracted with half maximal dose of noradrenaline. Concentrated OPP preparation was dissolved and diluted serially with buffer and added in cumulatively, directly to the bath. The change in tension was monitored using a computerised data acquisition system, and the extent of relaxation was calculated (BIOPAC Systems, Inc., Goleta, CA, USA).

The mesenteric arterial bed was prepared as described earlier^(^ ^20^ ^,^ ^22^ ^)^. The superior mesentery artery was cannulated and flushed with heparin saline, and the entire mesenteric bed including the intestinal tract was removed and the gut content was flushed out with saline. The preparation was mounted in a 50 ml organ bath chamber and continuously perfused with oxygenated Krebs–Henseleit medium. After 30 min of equilibration, tissue viability was assessed by cumulative intraluminal injection of various agonists (potassium chloride, noradrenaline). The pressure was raised by the addition of noradrenaline (half maximal dose) in the bath perfusate. Vasorelaxation response to the pharmacological agent acetylcholine (positive control) and OPP was determined by measuring the pressure reduction following intraluminal administration. Pressure changes were monitored using the MLT844 Physiological Pressure Transducer connected to a pressure amplifier (DA100C; BIOPAC Systems, Inc.) and a computer-based data acquisition system (MP100WSW High-performance data acquisition unit; BIOPAC Systems, Inc.).

### Teratological studies

The present study was designed to conform as closely as possible and, where applicable, to the United States Food and Drug Administration (US FDA) Department of Health and Human Services' Guidelines. The present study was also guided by a document of the FDA's Office of Food Additive Safety's Redbook 2000 (Toxicological Principles for the Safety Assessment of Food Additives). In conforming to the General Guidelines for Toxicology Studies' Good Laboratory Practice, the laboratory studies were conducted according to the US FDA Good Laboratory Practice regulations, issued under Part 58, Title 21 (Code of Federal Regulations). Female Albino Sprague–Dawley rats were obtained from the animal house facility at the Medical Faculty of University of Malaya (Kuala Lumpur, Malaysia). Ethical clearance was given by the Animal Care and Use Committee of the University of Malaya.

Female Albino Sprague–Dawley rats weighing about 200 g were mated, and the presence of the vaginal plug was taken to confirm pregnancy. Pregnant females were isolated in individual stainless-steel cages under the controlled conditions of the animal experimental unit. All rats were given standard rat chow. The controls were given water, while test rats were *ad libitum* given 1500 or 2400 mg/l gallic acid equivalent OPP as the sole drinking fluid from the day the vaginal plug was seen until the exact day of delivery (21–22 d).

The pregnant mothers were carefully monitored at least once daily. No abnormal behaviour was observed. The rats were weighed daily. The number of surviving pups at birth was noted, and any subsequent deaths or pups missing due to cannibalisation by the mother were also noted. After delivery, the rat pups and their mothers were kept in their stainless-steel cages. Standard rat chow and water were given to both the control and test mothers. The pups were kept with their mothers at all times except when observations and measurements were made. Except for those killed (by an overdose of chloral hydrate) for histological studies, the remaining pups were allowed to grow into adults, and the mating, feeding and other procedures were repeated twice (second and third generation).

Before necropsy and microscopic studies, the rat pups were observed from birth until 21 d after birth. Measurements or observations were recorded at birth, as well as 7, 14 and 21 d after birth.

The external parameters that were measured were body weight (g), nose–rump length (mm), tail length (mm) and tibial length (right and left sides) (mm). The physical examinations were carried out to assess exencephaly, eye defects (external), cleft palate, cleft lip, maxillofacial development, hydrocephalus, integument, locomotion, equilibrium and muscular tremors or abnormal movements.

The standard protocol for inspection of internal organs was carried out, and no obvious abnormalities or defects were observed, so it was concluded that no gross anomalies had developed. The organs were then harvested and processed for histopathology. Wax sections cut at 5 μm in thickness were mounted onto glass slides and stained with haematoxylin and eosin for light microscopy.

### Statistical analysis

Data were analysed using the Statistical Analysis System program (SAS Institute Inc., Cary, NC, USA). The experimental results are expressed as means and standard deviations, unless otherwise stated. For comparison of two groups, two-tailed unpaired Student's *t* test was performed, and significant differences between means were determined. For all outcomes, *P*< 0·05 was considered statistically significant.

## Results and discussion

### Oil palm phenolic components

**Fig. 1:**
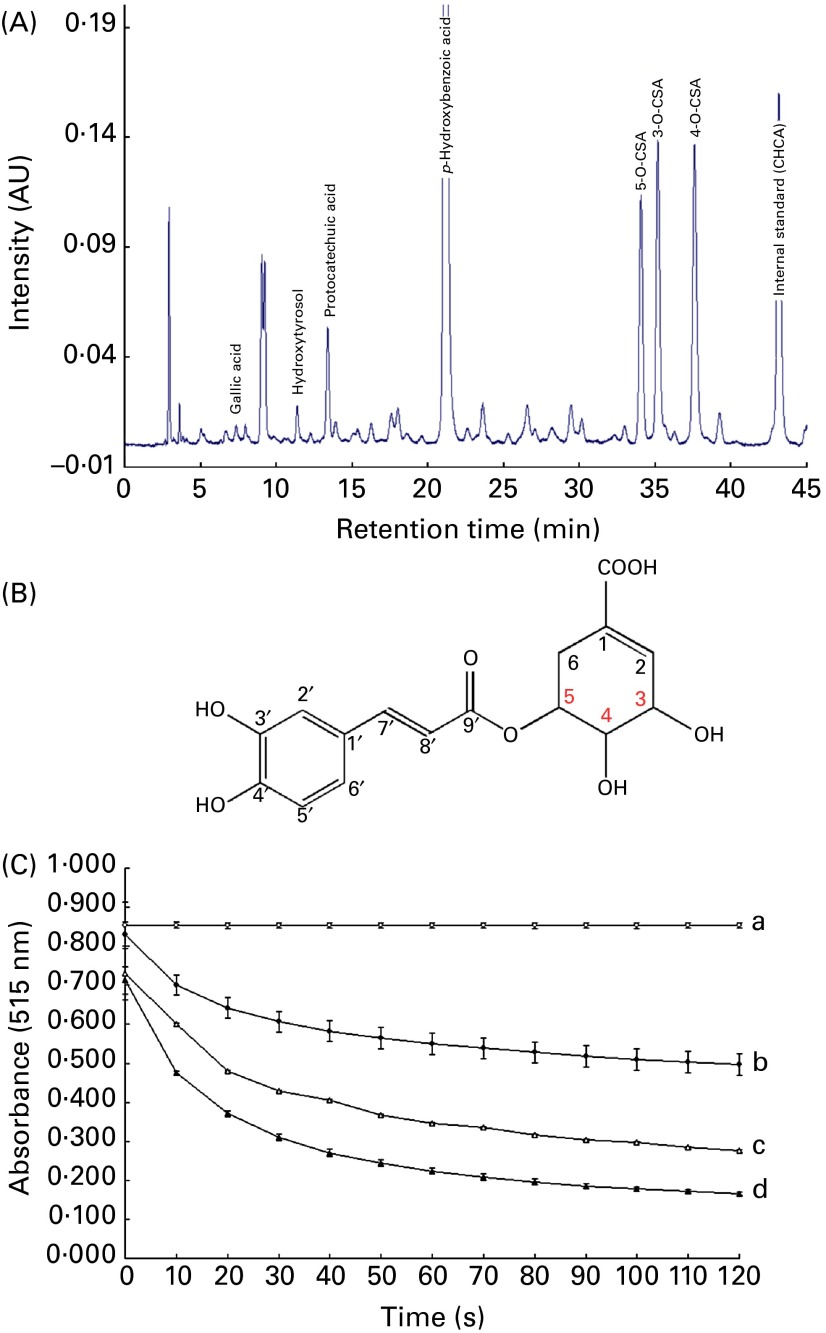
Components and antioxidant activity of oil palm phenolics (OPP). (A) HPLC profile of OPP indicating the presence of compounds such as hydroxytyrosol, *p*-hydroxybenzoic acid, protocatechuic acid and three isomers of caffeoylshikimic acid (CSA). α-Cyano-hydroxycinnamic acid (CHCA), an internal standard used in the HPLC analysis for quantification of the OPP components. (B) Structure of 5-*O*-CSA. (C) Antioxidant activity expressed as free radical scavenging activity (inhibition of 2,2-diphenyl-1-picrylhydrazyl). Lines with unlike letters were significantly different from one another (two-tailed unpaired Student's *t* test, *P*< 0·01, compared with a). –○–, Blank; –●–, 100 mg/l gallic acid equivalents (GAE); –△, 200 mg/l GAE; –▲–, 300 mg/l GAE. AU, arbitrary units.

Characterisation of the composition of OPP has been achieved using HPLC separation followed by MS and NMR. OPP contains numerous phenolic acids including caffeic acid, protocatechuic acid and *p*-hydroxybenzoic acid. We found that OPP also contains three isomers of caffeoylshikimic acid, a group of unique signature phenolics (Fig. 1(A) and (B)), as major components. The NMR data are given in the recently filed international patent^(^ ^23^ ^)^. The major contributors to the total phenolics are caffeoylshikimic acid at 10 800 (sd 2400) mg/kg, followed by *p*-hydroxybenzoic acid at 7000 (sd 1000) mg/kg (Table 1).

**Table 1:** Concentrations of major phenolic components in oil palm phenolics (OPP)* (Mean values, standard deviations and ranges)

	Concentration (mg/kg)
Phenolic compounds	Mean	sd	Range
Protocatechuic acid	600	100	400–800
*p*-Hydroxybenzoic acid	7000	1000	5300–8600
Caffeoylshikimic acid (total of three isomers)	10 800	2400	7700–15 100
Total major phenolics	18 400	2900	13 800–24 300
Gallic acid equivalents	18 200	1700	15 700–21 300

* Values are on a dry weight basis (mg of each major phenolic component for every kg of freeze-dried OPP) and represent triplicate analyses of OPP samples processed from the aqueous by-products obtained from six different Malaysian palm oil mills according to the methods described by Sambanthamurthi *et al.* ^(^ ^14^ ^)^.

The only other plant where caffeoylshikimic acid has been reported to be a major phenolic constituent is the date palm, *Phoenix dactylifera* ^(^ ^24^ ^)^. In this fruit, 3-*O*-caffeoylshikimic acid, also known as dactylifric acid, has been identified as one of the main enzymic browning substrates. The two isomers 4-*O*-caffeoylshikimic acid and 5-*O*-caffeoylshikimic acid are also known as isodactylifric and neodactylifric acid, respectively. Caffeoylshikimic acids represent one of the resistance factors of date palm roots towards *Fusarium oxysporum* ^(^ ^25^ ^,^ ^26^ ^)^. In addition, caffeoylshikimic acid has been identified as a minor constituent of yerba mate (*Ilex paraguariensis* L.)^(^ ^27^ ^)^ and vaccinium plants, lingonberry (*Vaccinium vitis-idaea* L.), bilberry (*Vaccinium myrtillus* L.) and hybrid bilberry (*Vaccinium*× *intermedium* Ruthe L.)^(^ ^28^ ^)^.

Shikimic acid and its esters are not commonly found in nature. It is likely that these compounds do not accumulate to an appreciable extent in most plants owing to their high metabolic turnover. For example, shikimic acid is central to the biosynthetic pathway for aromatic compounds such as tyrosine, tryptophan, phenylalanine and lignins, resulting in the rapid utilisation of this metabolite in plants. Caffeoylshikimic acid accounts for more than half of the total phenolic content of OPP, making it the largest known source of this compound.

### Antioxidant potential of oil palm phenolics

When the free radical scavenging activity of OPP was measured by DPPH in a standard assay^(^ ^9^ ^)^, OPP showed significant scavenging activity with a *t*_1/2_ (time to scavenge 50 % of the initial DPPH radicals) of less than 30 s at all concentrations tested (Fig. 1(C)). More than 75 % of the DPPH were scavenged by OPP at 100 mg/l gallic acid equivalent. The high efficacy and rate of free radical scavenging of OPP are generally indicative of protective antioxidant applications *in vivo*. Antioxidant activity of OPP is attributed to the ability to scavenge free radicals and donate hydrogen atoms. The antioxidant and radical scavenging activities increase with the degree of hydroxylation of the phenolic compound^(^ ^29^ ^)^. Caffeoylshikimic acid has four hydroxyl groups, and this would account for the potent antioxidant activity of OPP. Bearing in mind that OPP also has other phenolic acids present such as protocatechuic acid and *p*-hydroxybenzoic acid, these would also be expected to contribute to the antioxidant activity observed. Working together, these phenolic acids may also have a synergistic effect.

Pomace and other milling wastes have been reported to be rich in phenolics. However, these usually exist in the conjugated form with sugars and other moieties^(^ ^30^ ^,^ ^31^ ^)^. These conjugation reactions occur via the hydroxyl groups of phenolics, thus reducing the degree of hydroxylation and hence the antioxidant potential. Enzymatic hydrolysis of these glycosylated phenolics has been suggested as an attractive means of releasing free phenolics and hence, increasing their antioxidant potential^(^ ^30^ ^,^ ^31^ ^)^. Fortuitously, in the case of OPP, the sterilisation process during oil palm milling would hydrolyse glycosylated phenolics, releasing unconjugated phenolic acids such as caffeoylshikimic acids. OPP thus resembles coffee in that it consists mainly of phenolic acids and not flavonoids. Coffee consists of chlorogenic acids, which are a group of compounds comprising hydroxycinnamic acids, such as caffeic acid, ferulic acid and *p*-coumaric acid, linked to quinic acid to form a range of conjugated structures known as caffeoylquinic acids, feruloylquinic acids and *p*-coumaroylquinic acids^(^ ^32^ ^)^. Coffee has been reported to have higher antioxidant activities when compared with tea and cocoa on a cup-serving basis^(^ ^33^ ^)^.

### Protection against LDL oxidation

Oxidation of LDL has a pathogenic role in the development of atherosclerosis^(^ ^34^ ^)^. Uptake of oxidised LDL by macrophages and smooth muscle cells leads to the development of fatty streaks, a key event in early atherosclerosis. There is a positive correlation between the resistance of LDL to oxidation and the severity of coronary atherosclerosis in human subjects^(^ ^35^ ^)^. In the present study, OPP dose-dependently inhibited the Cu-mediated oxidation of human LDL *in vitro*. It increased the lag time of conjugated diene formation from 67 (control) to 74, 103 and 121 min at concentrations of 0·25, 0·50 and 1·00 mg/kg gallic acid equivalent, respectively, implying potential efficacy against atherogenesis. A commercial preparation of pure catechin showed a similar effect (Table 2). The results are consistent with other investigations carried out using wine, cocoa and green tea, showing positive correlations between inhibition of LDL oxidation and the amount of total phenolic compounds^(^ ^36^ ^–^ ^38^ ^)^. Phenolics prevent LDL oxidation *in vitro* by scavenging radical species or sequestering metal ions^(^ ^39^ ^,^ ^40^ ^)^. This protection by phenolics indicates a decreased risk of CVD when changes in the susceptibility and extent of LDL oxidation are implicated as important causative factors.

**Table 2:** Effect of oil palm phenolics (OPP) on copper-mediated oxidation of human LDL* (Mean values and standard deviations, *n* 6 (LDL preparations))

	Lag time (min)
Test compounds	Mean	sd
LDL (control)	67	12
OPP (0·25 mg/kg)	74	9
OPP (0·50 mg/kg)	103†	14
OPP (1·00 mg/kg)	121†	7
Catechin (0·25 mg/kg)	87	13
Catechin (0·50 mg/kg)	104†	10

* All oxidations were conducted in triplicate and averaged for each LDL preparation.

† Mean values were significantly different from LDL (control; *P*< 0·05).

### Aortic ring and mesenteric vascular bed vascular relaxation

Isolated vascular preparations such as the aortic ring and perfused mesenteric vascular bed have routinely been used as reliable *in vitro* methods to investigate the vascular actions of therapeutic agents as well as potential bioactives. This model allows the investigation of potential vasodilatory effects including an assessment of the role of vascular endothelium in mediating potential benefits. Endothelial-derived NO induces vasodilation by diffusing across the endothelium into the adjacent smooth muscle, causing the smooth muscle to relax and dilate. NO is produced from l-arginine in a reaction catalysed by endothelial NO synthase.

**Fig. 2:**
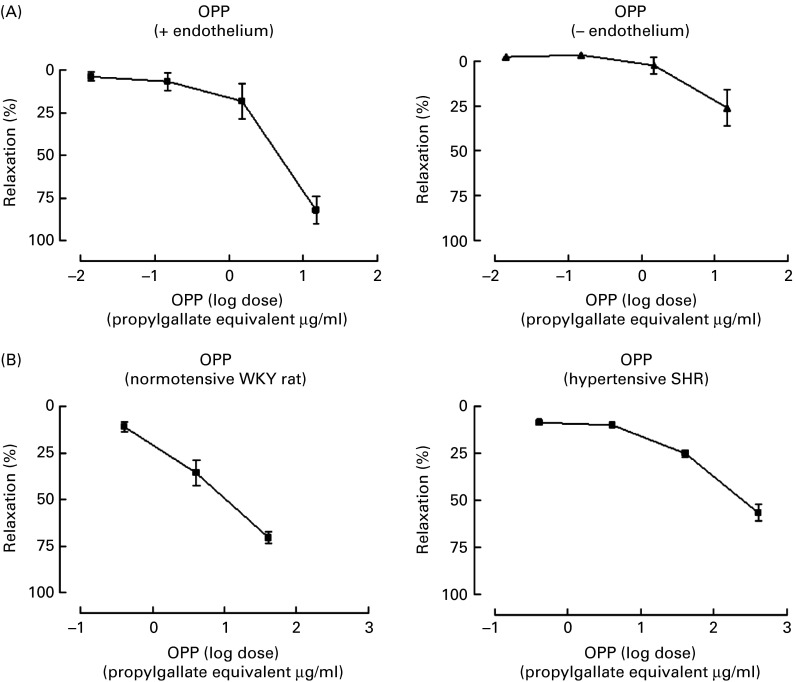
Vascular relaxation actions of oil palm phenolics (OPP). (A) Responses following cumulative addition of OPP to endothelium intact and denuded aortic rings from normotensive rats. (B) Responses following intraluminal administration of OPP to a perfused mesenteric vascular bed from normotensive and spontaneously hypertensive rats. Values are means, with their standard errors represented by vertical bars (*n* 6). WKY, Wistar Kyoto; SHR, spontaneously hypertensive rat.

To assess for possible vascular protection effects, we used aortic ring and mesenteric vascular bed preparations in the present study. OPP dose-dependently promoted vascular relaxation in endothelium-intact isolated aortic rings (conductance vessels) and in a perfused mesenteric vascular bed (resistance vessels) (Fig. 2). These results indicate that the vascular relaxation induced by OPP was mediated via endothelial NO, a major endogenous vasodilator involved in maintaining cardiovascular homeostasis.

In physiological terms, the aorta does not contribute to peripheral vascular resistance. However, changes in vascular reactivity and compliance in larger vessels (stiffness–elasticity) may influence vascular flow characteristics. Indeed, a large body of evidence indicates that different plant-based compounds, which showed positive results in the aortic ring preparation (e.g. grape and wine polyphenols, cocoa and coffee phenolics, soya isoflavones), have also been found to produce vasodilation and improve vascular function in whole animals and in human subjects^(^ ^41^ ^–^ ^47^ ^)^.

The mesenteric vascular bed is an important contributor to systemic vascular resistance, and compared with the larger vessels such as the aorta, contractions of smaller arteries are more relevant to blood pressure regulation. It is known that both an increased cardiac output and an increased vascular resistance in the peripheral circulation can lead to hypertension. Therefore, tissue preparations, which measure vascular function in resistance vessels, can be regarded as more relevant for studies on blood pressure regulation and hypertension. In the perfused mesenteric preparation, the changes in intraluminal pressure development due to the constriction of resistance arteries (peripheral circulation) are measured. The present findings that OPP dose-dependently promoted relaxation in both larger vessels (aorta) and resistance vessels suggest that this mix of phenolics may be effective in lowering blood pressure in the whole animal.

### Teratological studies

**Fig. 3:**
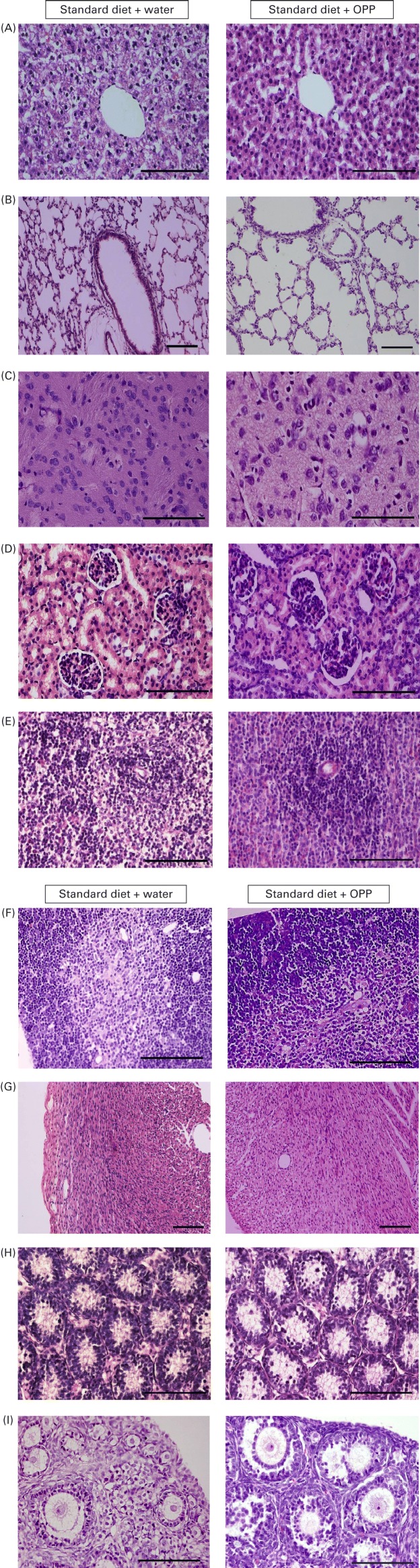
Representative haematoxylin and eosin-stained tissue slices from major organs of third-generation rats viewed under a light microscope. (A) Liver, (B) lung, (C) brain, (D) kidney, (E) spleen, (F) thymus, (G) heart, (H) testis and (I) ovary. Oil palm phenolics (OPP) did not show teratogenic effects. Scale bars represent 100 μm.

Administration of OPP to rats did not affect the well-being of the animals, and no signs of OPP-induced toxicity were observed, as determined by gross morphological examination of major organs. We also ruled out teratogenic effects by monitoring Sprague–Dawley rats for three generations. There were no observable developmental birth defects or congenital anomalies in the offspring of rats supplemented with OPP at both 1500 and 2400 mg/l gallic acid equivalents in their water supply for all three generations tested. There was no significant difference in the number of surviving offspring. Physical examinations including macroscopic observations for exencephaly, external eye defects, cleft palate/lip, maxillofacial development, hydrocephalus, integument, locomotion, equilibrium and muscular tremors did not reveal any abnormalities caused by OPP. The growth milestones of the offspring were also not significantly different from the controls for the three generations studied. The histology of all organs tested, including liver, lungs, brain, kidneys, spleen, thymus, heart, testes and ovaries, was normal (Fig. 3). The weights of the organs tested were also not significantly different.

### Conclusions

The burden of chronic diseases is rapidly increasing worldwide. Oxidative stress is a unifying mechanism in the aetiology of chronic diseases. As such, dietary antioxidants such as widespread plant phenolics may be important for the prevention and treatment of chronic diseases. The discovery that the aqueous stream of the oil palm milling process contains potent phenolics raises the possibility that a major disposal liability can be turned into beneficial use against chronic disease processes. It has been established that phenolic compounds in wastewaters from oil milling industries are toxic to plants and micro-organisms. Removal of phenolic compounds from these wastewaters has been shown to attenuate toxicity^(^ ^48^ ^)^. Thus, the palm oil mill effluent discharged following removal of phenolics is expected to be less toxic. The present data indicate that caffeoylshikimic acids, which are rare in nature, are the major components of OPP. The present study also confirmed that OPP displays antioxidant properties with potentially far-reaching physiological effects without evidence of toxicity. In conclusion, OPP from the aqueous stream of the palm oil milling process has significant protective bioactivities against CVD, without causing toxicities and teratological effects in pre-clinical models. This discovery makes it possible to turn a major disposal liability into a unique, potentially valuable resource for pharmaceutical and healthcare functions.
